# Modulation of Insulin Sensitivity by Exercise Training: Implications for Cardiovascular Prevention

**DOI:** 10.1007/s12265-020-10057-w

**Published:** 2020-07-31

**Authors:** Guido Iaccarino, Danilo Franco, Daniela Sorriento, Teresa Strisciuglio, Emanuele Barbato, Carmine Morisco

**Affiliations:** Department of Advanced Biomedical Science, Federico II University, Via S. Pansini n. 5, 80131 Naples, Italy

**Keywords:** Physical training, Resistance exercise, Cardiovascular risk, Insulin resistance, Insulin signaling, Essential hypertension, Diabetes, Obesity, Metabolic syndrome

## Abstract

The beneficial effects of physical activity on the cardiovascular system nowadays have achieved the relevance of clinical evidence. In fact, several studies have documented the benefits of exercise training in the prevention of the cardiovascular risk. Abnormalities of insulin signaling transduction account for the impairment of insulin sensitivity and development of insulin resistance, which, in turn, is responsible for the enhancement of cardiovascular risk. Insulin sensitivity is related to the degree of physical activity, and physical training has been shown to ameliorate insulin action in insulin-resistant subjects. This effect is mediated by the improvement of the molecular abnormalities that are responsible of the insulin resistance, contributing in this way to restore the physiological insulin sensitivity. However, it should be underlined that mechanisms that account for this phenomenon are extremely complex and still unclear. Further studies are required to better clarify the molecular basis of the exercise-evoked improvement of insulin signal.

**Figure:**
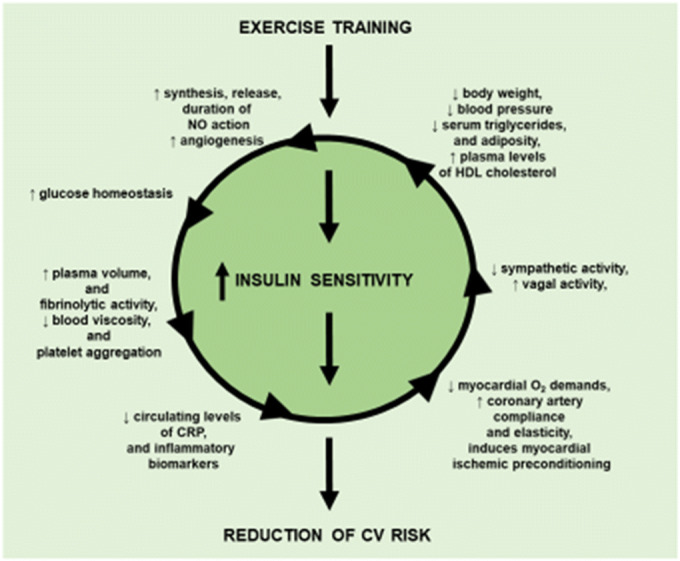
Graphical abstract

Graphical abstract

*“…* a normal person should exercise seven hours a week. If could not exercise an hour everyday, make up the difference on the weekend”.

*Dr. Paul Dudley White 1955*

The beneficial effects of physical activity on the cardiovascular system were a simple intuition of the father of modern cardiology in the middle of the last century that nowadays has achieved the relevance of clinical evidence. In fact, there are incontrovertible data derived from observational studies and controlled trials that show how regular physical exercise (training) both aerobic (continuous walking, jogging, and cycling) and resistance exercise (weights), alone or as a part of lifestyle modification, is able to prevent or, at least, delay the development of the major determinants of cardiovascular (CV) risk, as well as to ameliorate the prognosis of CV diseases. Considering that in the western countries a sedentary lifestyle is an endemic behavior, and can be considered a leading cause of death and disability from CV diseases [1], physical training is highly recommended by the World Health Organization [2] as well as by the guidelines from various scientific societies [3–7], and at the same time it is considered a key strategy for CV prevention by several healthcare systems [8–11].

## Exercise Training and CV Risk Factors

Several studies documented the benefits of exercise training in the prevention of obesity, metabolic and hemodynamic disorders as diabetes, metabolic syndrome (MS), dyslipidemias, and hypertension.

### Exercise and Obesity

Obesity is considered an independent determinant for heart failure [12], left ventricular hypertrophy [13], and significantly accounts for the increase of CV risk. Physical training represents a key component of behavioral interventions for the prevention and management of overweight and obesity. The favorable effects of exercise on subjects with overweight or obesity have been reported by several systematic reviews [14, 15]. However, all these analyses reported only a marginal effect on weight reduction (≈ 1–5 kg), when exercise was used alone as a weight loss intervention, in comparison with no treatment. In addition, it has also been documented that high intensity is more effective than moderate or light intensity exercise in inducing weight loss [16]. Notably, in obese subjects, despite the modest effects on weight loss, exercise training induces several important beneficial effects on blood pressure (BP), metabolic profile, and quality of life [17]. In addition, physical activity has an important role in weight loss maintenance [18]. A meta-analysis that compared the outcome at 1 year of follow-up of the combination of diet + exercise with diet alone, in the weight loss maintenance, showed that combined treatment induced a 20% greater weight loss compared with diet alone [19]. These data were consistent with the results of the National Weight Control Registry [20], who demonstrated in a 10-year observational study that long-term weight loss maintenance requires the combination of leisure-time physical activity (LTPA), hypocaloric diet, associated with behavioral changes like self-weighing frequency. The results of different cross-sectional and prospective studies on the role of physical training in the maintenance weight loss were implemented by the American College of Sports Medicine that stated “….after weight loss, weight maintenance is improved with physical activity >250 min/weak” [21].

### Exercise and Diabetes

The more convincing and extensive results of the exercise benefits on CV risk come from interventional trials that included subjects at high risk for the development of diabetes. For instance, The Chinese Da Qing Impaired Glucose Tolerance and Diabetes Study [22] that randomized 577 subjects with impaired glucose tolerance (IGT), in four groups: control group or to one of three active treatment groups: diet only, exercise only, or diet plus exercise, found that in the control group, the cumulative incidence of diabetes at 6 years was 67.7%, while in diet only, exercise only, or diet plus exercise groups was 43.8%, 41%, and 46%, respectively, with a reduction for the exercise activity intervention of the adjusted risk for development of diabetes of 42%. These results were confirmed by several studies in different ethnicities [23–25]. Interestingly, two longitudinal studies achieved similar results: The Finnish Diabetes Prevention [26] and the Diabetes Prevention Program [27] showed, in non-diabetic persons at elevate risk for diabetes, that a lifestyle-modification program associated with a regular physical activity is able to reduce the incidence of the diabetes by 58%. However, it should be underlined that almost all studies that demonstrated in subjects with IGT, the ability of exercise to reduce or delay the incidence of diabetes, included also hypocaloric diet. Therefore, in light of these studies, it is possible to conclude that diet and exercise exert a synergic effect in the prevention of diabetes. Thus, further studies are needed to evaluate whether physical activity alone influences the risk of diabetes [28]. However, it should be underlined that there are robust evidences clearly demonstrating that greater physical activity level confers higher protection against the risk to develop type 2 diabetes. In fact, a post hoc analysis of the Finnish Diabetes Prevention Study demonstrated a further reduction of the relative risk of diabetes by 63–65%, in those subjects that increased their LTPA from moderate-to-vigorous or strenuous, during 4.1 years of follow-up period [29].

These results were consistent with the Nurses’ Health Study [30], a large prospective cohort study that analyzed 70.102 women, aged from 30 to 55 years, free from diabetes, followed up for 8 years, and demonstrated that higher LTPA level, in terms of both duration and intensity, was associated with decreased risk to develop type 2 diabetes. It is noteworthy that the relationship found between the degree of exercise and the risk of diabetes remained significant also after adjustment for the body mass index, highlighting the importance of physical activity level in the prevention of diabetes, independently from other factors. The favorable effects of exercise training are limited not only to the prevention, but also to the control of the prognostic determinants of the diabetes, such as the glycaemia, blood pressure, and lipid profile. The main goal of the treatment of diabetes is to reduce the hyperglycemia. Glycosylated hemoglobin (HbA1c) is a biomarker of long-term glycemic control. In diabetic patients, exercise training was shown to decrease plasma levels of HbA1c. Unfortunately, this finding was obtained in small sample sized studies with limited statistical power. Therefore, Boulè et al. [31] performed a meta-analysis of 14 controlled clinical trials with a follow-up duration of more than 8 weeks. The pooled data confirmed that exercise training significantly reduced the plasma levels of HbA1c (− 0.66%; *p* < 0.001); in addition, the meta-regression analysis demonstrated that this effect was independent from changes in body weight. The magnitude of this reduction was similar to that achieved between the conventional- and intensive-glucose lowering therapy in the United Kingdom Prospective Diabetes Study (UKPDS). To note, that tight control of glycaemia is associated with a reduction of diabetes-related major clinical events.

Furthermore, it has been also documented that exercise training reduces systolic blood pressure (SBP) in patients with diabetes (by a magnitude of 4 mmHg) [32]. Although this reduction was found not statistically significant in comparison with the control patients, it is clinically relevant because it has been documented that a reduction of CV risk is achieved also with modest decreases in blood pressure values. Finally, it has been reported that dyslipidemia is a feature of diabetes that is characterized by increased circulating levels of triglycerides and oxidized low-density lipoproteins (LDL), associated with a decreased HDL. Although it is widely accepted that exercise training improves the metabolic profile, there are only few studies that demonstrate the benefits on lipid profile in diabetic patients. The Action for Health in Diabetes (The Look AHEAD Trial) [33] showed that 1-year of exercise training associated with hypocaloric diet decreased circulating levels of LDL and triglycerides, and increased HDL.

Interesting albeit speculative data suggest that exercise training might be associated with lower mortality in diabetes. Wei et al. [34] found in a prospective cohort study in 1263 diabetic subjects followed for 12 years that low cardiorespiratory fitness and physical inactivity independently increased the risk of all-cause mortality. Similar results were reported by Church et al. [35] who showed in 2196 diabetic subjects an inverse relationship between cardiorespiratory fitness and mortality.

### Exercise and Metabolic Syndrome

The metabolic syndrome (MS) is a cluster of metabolic, hemodynamic, and anthropometric abnormalities and is a precursor of CV diseases. It has been reported an inverse relationship between prevalence of MS and physical activity level [36, 37]. In addition, as for the diabetes, also for the MS, it has been documented by both observational and intervention studies that, in subjects at high risk, the exercise training reduces the incidence of this condition and of its single components [38]. The Look AHEAD study [39] evaluated 5145 overweight or obese patients with type 2 diabetes whether lifestyle intervention would decrease CV morbidity and mortality. The subjects were randomly assigned to an intensive lifestyle intervention that included weight loss through decreased caloric intake and increased physical activity (intervention group) or to receive diabetes support and educational program (control group). This study showed that the intensive lifestyle intervention did not reduce the incidence of CV events, however produced many beneficial effects as a greater reduction in weight and waist circumference, and in HbA1c, and a greater initial improvement in fitness and all CV risk factors, except low-density lipoprotein (LDL) cholesterol. Consistently, other studies confirmed the favorable effects of exercise on components of the MS. For instance, it has been documented, in a randomized controlled trial [40], that analyzed 51 men and 53 women aged 55–75 years with untreated SBP or diastolic blood pressure (DBP) that exercise training increased aerobic and strength fitness, reduced total and abdominal obesity, and increased lean body mass, as well as ameliorated cholesterol HDL and SBP. At baseline, MS was present in 43.2% of the studied population; at 6 months, 17.7% and 15.1% in the exercise and in the control group, respectively, no longer had the criteria of MS.

In clinical practice, it is also relevant the type of exercise needed to prevent the single components of MS. At this regard, a systematic review compared in subjects with MS without diabetes, a condition that represents the great majority of the subjects with MS, the aerobic vs. resistance exercise on the occurrence of single components of MS [41]. This analysis included 11 studies, with 16 interventions (12 aerobic, 4 resistance) and 588 subjects. The analysis showed that aerobic exercise significantly improved all single components of MS like waist circumference, fasting glucose, high-density lipoprotein (HDL) cholesterol triglycerides, and DBP. On the contrary, resistance exercise did not evoke any significant effect; however, it should be considered that this result could be due to limited data available.

### Exercise and High-Density Lipoprotein

The favorable effects of physical training on lipid profile and lipoproteins are well documented. Since the low HDL-cholesterol levels increase the risk of coronary heart disease and atherosclerosis, it is particularly relevant in terms of CV prevention the capability of physical training to raise levels of HDL cholesterol [42]. In this regard, a randomized study reported in healthy older adults that a 2-year period of aerobic physical training enhanced the plasma levels of HDL-cholesterol level by 3–9% [43]. Interestingly, this response was dependent by the intensity and frequency of the physical activity resulting more pronounced in those subject assigned to a frequent, low-intensity exercise. The relevance of exercise characteristics such as intensity, caloric expenditure, and duration in the regulation of HDL cholesterol was further confirmed by Kraus et al. who demonstrated that beneficial effects on the HDL cholesterol were related to the amount of physical activity rather than the intensity of exercise or improvement in fitness [9]. These data were confirmed by a meta-analysis [44] that included 35 studies and 1404 individuals that showed that exercise duration per session was the most important determinant of HDL-cholesterol increase. Interestingly, it has been raised the hypothesis that exercise is able also to modulate HDL-cholesterol metabolism and function; however, this needs to be more extensively investigated [45].

In general, there is a large consensus that aerobic exercise training is capable of preventing the cardio-metabolic risk by increasing HDL cholesterol and decreasing the incidence of type 2 diabetes and MS.

### Exercise and Blood Pressure

In addition to the metabolic effect, there is also a general agreement that either aerobic exercise or resistance exercise exerts a favorable action on BP homeostasis in normal subjects and in patients with arterial hypertension. However, it should be noted that this notion for several decades did not receive any relevance and remained a weak suggestion for the management of hypertension. The first evidence that exercise can ameliorate BP control comes from the HARVEST Trial [46]. This was a cross-sectional study that demonstrated, in young hypertensive patients who had never been treated for hypertension, and categorized as exercisers and non-exercisers, that physically active men had a lower 24-h and daytime DBP compared with the inactive men, even after adjustment for age, body mass index, alcohol intake, and smoking status. The first randomized controlled study that evaluated the effects of exercise on BP was the PREMIER Clinical Trial [47]. This study analyzed 810 subjects with high-normal BP or stage 1 hypertension, who were not taking antihypertensive medications, the effects of the combination of the DASH diet with three different and well-established lifestyle recommendations for lowering BP: weight loss, exercise, and restriction of sodium and alcohol. At 6 months, the combination of DASH diet + lifestyle recommendation decreased SBP and DBP both in hypertensive (− 6.1/− 3.6 mmHg) and in normal (− 3.1/2.0 mmHg) subjects. The results of this large randomized interventional study strongly supported the concept that multiple lifestyle changes are able to lower BP at same extent of some drugs.

The effects of exercise on both office and ambulatory BP have been investigated in two meta-analyses. In particular, one meta-analysis [48] explored the effects of different types of exercise in 5223 participants (3401 exercise and 1822 control) showed that all types of physical activities were able to reduce both SBP and DBP. The magnitude of office BP decrease, evoked by endurance training, was significantly higher in hypertensive subjects (− 8.3/− 5.2 mmHg for SBP and DBP, respectively) in comparison with pre-hypertensive subjects (− 2.1/− 1.1 mmHg, for SBP and DBP) and normal individuals (− 0.75/− 1.1 mmHg for SBP and DBP). Notably, dynamic resistance training was particularly effective to reduce BP (− 4.0/− 3.8 mmHg, for SBP and DBP, respectively) in pre-hypertensive subjects compared with patients with hypertension or normal individuals. Similarly, it has been shown that endurance training favorably affects also ambulatory BP. Another meta-analysis [49] investigating 633 participants (394 exercise participants and 239 control participants) showed that aerobic endurance exercise induced a significant reduction in day-time SBP and DBP (− 3.2 and – 2.7 mmHg, respectively), while it did not affect the night-time BP. Unfortunately, only few studies have explored the effect of resistance exercise on the BP. The results of these studies show only a minimal impact on BP [50]. However, the beneficial effects on the pathophysiological mechanism of hypertension of the high-intensity exercise allow to speculate that this form of exercise may have a favorable impact on hypertension [51]. In summary, the statement of the American College of Sports Medicine [52] that recommended to perform dynamic aerobic endurance training for at least 30 min daily, preferably supplemented with dynamic resistance exercise for the primary prevention and control of hypertension, can be still considered valid. The beneficial effects of physical training not only are limited to the prevention and management of hypertension but also include the reduction of mortality. In general, there is the evidence that LTPS is inversely associated with all-cause mortality in both men and women [53]. Moreover, an analysis [54] that evaluated the results of 6 prospective longitudinal studies that included 48,448 male and 47,625 female hypertensive patients showed that physical activity decreased the risk of cardiovascular and/or all-cause mortality at least by 16%.

**Fig. 1:**
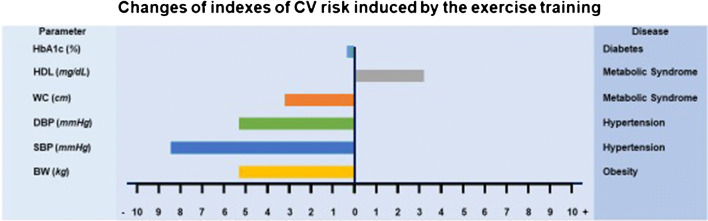
The effects of exercise training on the conventional determinant of CV risk. HbA1c: glycosylated hemoglobin, HDL: high-density lipoprotein, WC: waist circumference, DBP: diastolic blood pressure, SBP: systolic blood pressure, BW: body weight

The impact of exercise training on the conventional determinants of the CV risk is summarized in Fig. 1.

## Effects of Exercise on Insulin Resistance

Insulin exerts the control on glucose, lipid, and protein metabolism acting on the skeletal muscle, liver, and white adipocytes. In particular, in the skeletal muscle, insulin stimulates the glucose uptake and the glycogen synthesis; in the liver, inhibits the gluconeogenesis, and promotes both the glycogen synthesis and the lipogenesis; in the withe adipocytes, decreases the lipolysis and enhances both the glucose uptake and the lipogenesis. Insulin resistance can be defined as a less than expected biologic response to a given concentration of the hormone. The direct consequence of this phenomenon is the raising of circulating insulin levels which are necessary to maintain the metabolic homeostasis. Therefore, hyperinsulinemia is the first hallmark of reduced insulin sensitivity. Several physiological conditions are able to impair the insulin action like hypercaloric diet, weight gain, inflammatory states, sympathetic stimulation, and hyperinsulinemia [55]. To note that decreased insulin sensitivity is a reversible phenomenon, however, sustained reduced responsiveness to the insulin triggers a vicious circle that, finally, amplifies the resistance to the hormone and accounts for the development of diabetes and/or other determinants of CV risk. Since the majority of somatic cells express the insulin receptor and share the early steps of insulin signal transduction, insulin has a pleiotropic action and plays a key role not only in the regulation of metabolic homeostasis, but also in the ion and amino acid transport, in the cell proliferation, differentiation, and death, in the vascular function and inflammation, and in the neuro-hormonal activation. Abnormalities of insulin signaling transduction account for the development of insulin resistance which plays a mechanistic role in the pathogenesis of several CV risk factors such as diabetes, metabolic syndrome, hypertension, atherosclerosis, obesity, and non-alcoholic fatty liver disease (NAFLD). In addition, insulin resistance accounts also for the enhanced CV risk, and has been identified as an independent factor for the incidence of CV diseases including coronary artery disease, heart failure, and stroke. Moreover, insulin resistance negatively affects the clinical outcomes of CV and cerebrovascular diseases.

In general, the insulin sensitivity is related to the degree of physical activity, and physical training has been shown to ameliorate insulin effects in insulin-resistant subjects. Interestingly, it has been documented that intensity and volume of exercise program have different effects on insulin sensitivity. In particular, Houmard et al. have demonstrated [56] in sedentary, overweight/obese subjects randomly assigned to three different 6-month training programs (low-volume/moderate-intensity, low-volume/high-intensity, high-volume/high-intensity) that in comparison with the control group, all training programs enhanced the insulin sensitivity index. However, those exercise programs that incorporated ∼ 170 min of exercise/weekly improved insulin sensitivity (≈ 85%) more than a program utilizing ∼ 115 min of exercise/weekly (≈ 40%), suggesting that exercise duration rather than intensity plays a role to improve insulin sensitivity.

Insulin resistance is the principal pathogenic mechanism of MS. In subjects with MS, compared with normal healthy individuals, it has reported [57] lower circulating levels of the biomarkers that are expression of nitric oxide (NO) production such as whole blood nitrite, plasma nitrite, and plasma cyclic guanosine mono-phosphate (cGMP), and, at the same time, increased oxidative stress and asymmetrical dimethylarginine (an endogenous NO synthase inhibitor). This condition was reverted by 3-month exercise training, which restored the circulating levels of whole blood nitrite and cGMP, and decreased both oxidative stress and the circulating levels of asymmetrical dimethylarginine, suggesting that exercise increases NO synthesis. Studies on muscle biopsies in sedentary obese subjects elucidated [58] the molecular mechanisms of exercise-induced enhanced production/stabilization of NO, documenting that exercise training significantly enhanced the expression of endothelial nitric oxide synthase (eNOS) content in muscle terminal arterioles and capillaries, as well as eNOS ser^1177^ phosphorylation. Interestingly, these changes were associated with an improvement of indexes of insulin sensitivity. These studies do not clarify whether exercise training-induced enhanced NO bioavailability is responsible for enhanced insulin sensitivity or vice versa [59]. As abovementioned, insulin resistance is a feature of obesity and it is associated with enhanced circulating levels of mediators of vascular inflammation such as leptin, adiponectin, interleukine- (IL) 6, and tumor necrosis factor- (TNF) α. The adipose tissue substantially contributes to the production and release of these factors. It has been reported [60] in obese subjects that 24 weeks of moderate- to high-intensity physical training reduced the circulating levels of markers of subclinical inflammation such as C-reactive protein (CRP), resistin, and concomitantly improved insulin resistance. The beneficial effects of exercise on both insulin sensitivity and vascular inflammation have been confirmed in different studies and meta-analyses. In particular, one meta-analysis [61] that included 7487 participants showed that exercise significantly decreased levels of fasting insulin, homeostatic model assessment–insulin resistance (HOMA), and HbA1c, as well as the levels of the mediators of vascular inflammation such as leptin, fibrinogen, and angiotensin II.

There is a close relationship between the sympathetic nervous system and insulin sensitivity. Dysregulation of the sympathetic nervous system, resulting in its overactivity, contributes to development of insulin resistance [62]. Insulin stimulation increases the sympathetic nervous system activity by affecting the baroreflex sensitivity, and acting centrally [63]. There is consistent evidence that exercise training improves the baroreflex control of the sympathetic nervous system [64, 65] and reduces the sympathetic activity [66], also in those conditions characterized by insulin resistance as MS [67] and hypertension. Interestingly, it has been demonstrated in obese subjects that exercise training program improves cardiac autonomic modulation in response to an oral glucose load, suggesting that physical training may improve autonomic responses by decreasing insulin resistance [68]. Since insulin plays a role in the regulation of peripheral vascular resistances by blunting the sympathetic-induced vasoconstriction, insulin resistance is characterized also by the impairment of the control of vascular tone. Thus, the improvement of both insulin sensitivity and sympathetic activity induced by the exercise training ameliorates the neuro-metabolic control of the vascular tone. The contribution to the improvement of vascular function induced by the exercise training is a complex and multifaceted phenomenon that includes also the angiogenesis of capillaries, remodeling and enlargement of arteries and arterioles and arterio-genesis [69]. Together these adaptations improve the microcirculation blood flow in the skeletal muscle, and consequently contribute to increase insulin-induced glucose uptake [70].

Many of the beneficial effects on the pathogenic mechanisms responsible for the CV risk factors evoked by the training activity such as the increase of the synthesis, the release and the stability of nitric oxide, the reduction of the plasma levels of the mediators of vascular inflammation, the raise of the plasma levels of HDL cholesterol, the improvement of control and the anatomy of peripheral vasculature, and the increase of energetic efficiency are associated with the improvement of insulin sensitivity; however, it is still undefined whether these changes are the consequences or the cause of improvement of insulin sensitivity. From a clinical point of view, this is not a trivial question since it is clear that altogether these effects act in concert to reduce the CV risk.

## Exercise-Induced Modulation of Insulin Signaling

**Fig. 2:**
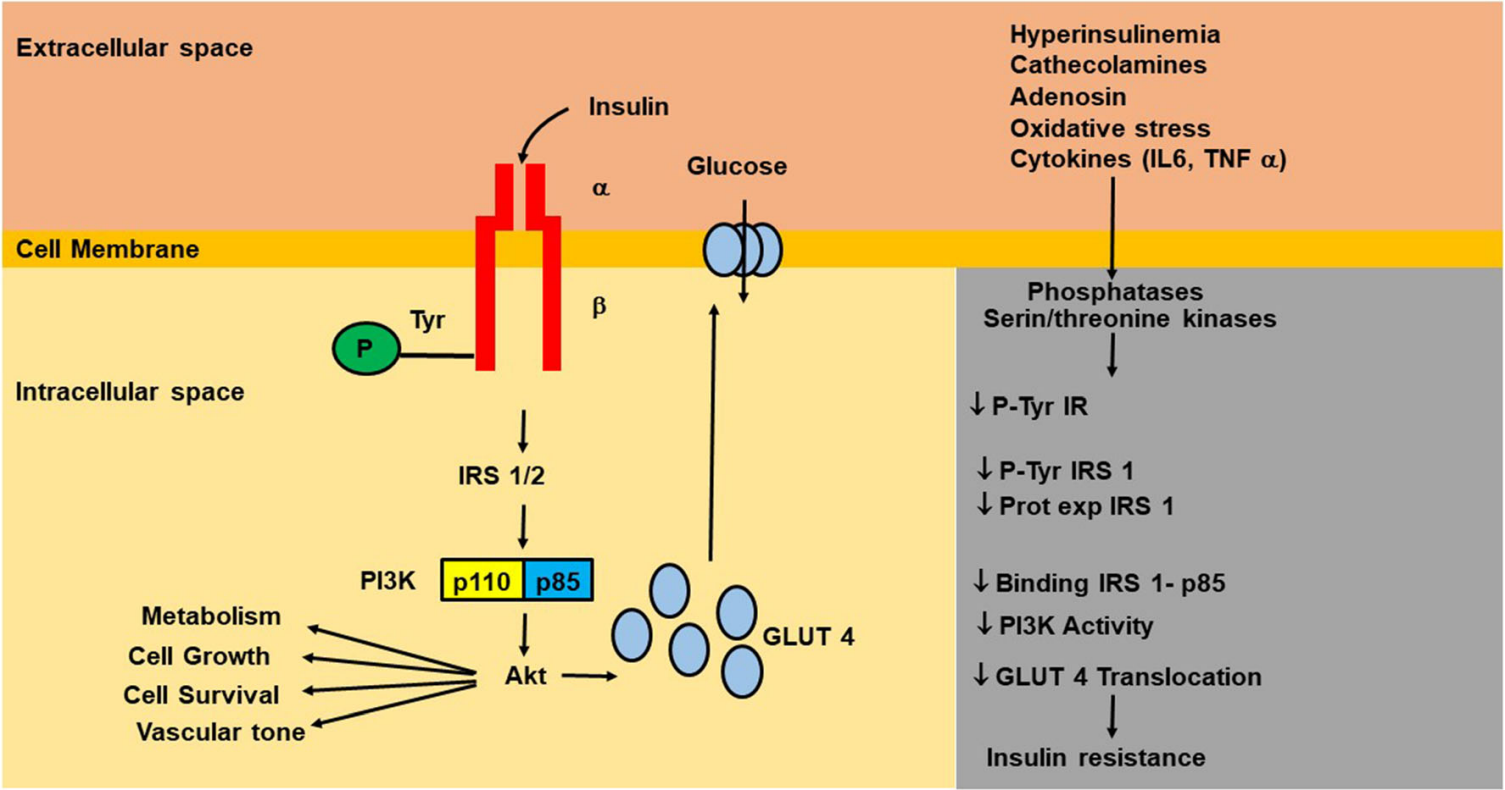
Schematic representation of early steps of insulin signaling, and the principal mechanisms that account for insulin resistance. Binding of insulin to the α-subunit of its own receptor induces the autophosphorylation of tyrosine residues of β-subunit which, in turn, induces the tyrosine phosphorylation of IR substrates (IRS), IRS-1, and IRS-2. The binding of phosphorylated IRS1/2 to the regulatory subunit p85 of phosphoinositide-3 kinase (PI3K) activates the catalytic subunit p110, which, in turn, phosphorylates/activates the serine/threonine kinase Akt (called also PKB), which stimulates the glucose uptake through the translocation of the major glucose transporter GLUT-4 to the plasma membrane. In addition, AKT promotes cell survival and cell growth, and is involved in the regulation of vascular tone and metabolic homeostasis. Insulin resistance evoked by oxidative stress, neuro-hormonal stimulation, hyperinsulinemia, and etc. may be due to defect(s) of downstream signaling components, such as receptor structure, number, binding affinity, and/or signaling capacity. P: phosphorylation, Tyr: tyrosine, IR. insulin receptor, IL-6: interleukine-6, Pro Exp: protein expression, TNF: tumor necrosis factor

Abnormalities of insulin signaling are responsible of insulin resistance. Insulin evokes its action on the target organs through the phosphorylation of a transmembrane-spanning tyrosine kinase receptor, the insulin receptor (IR). The binding of insulin to the α-subunit of its receptor activates the tyrosine kinase of the β-subunit of the receptor, leading the own autophosphorylation, which, in turn, induces the tyrosine phosphorylation of IR substrates (IRS), IRS-1, and IRS-2. The phosphorylation/activation of these substrates evokes the activation of phosphoinositide 3-kinase (PI3K), which, in turn, stimulates the activation of the main downstream effector Akt (also called protein kinase B) and protein kinases C subtypes λ and ζ. Akt is serine/threonine kinase, which stimulates the glucose uptake through the translocation of the major glucose transporter GLUT-4 to the plasma membrane (Fig. 2). Furthermore, Akt inactivates, by phosphorylation, the glycogen synthase kinase 3 (GSK-3), and this enzyme inhibits glycogen synthase which is a major regulator of glycogenic process, therefore, facilitating the glycogenesis.

Insulin resistance may be due to defect(s): (1) at some point before insulin binding to its receptor, (2) at IR level, and/or (3) defects in downstream signaling components. Defects of the IR that may contribute to insulin resistance include abnormalities in receptor structure and number, binding affinity, and/or signaling capacity. Several factors including the oxidative stress, the neuro-hormonal stimulation, and the hyperinsulinemia have been described as responsible for the inhibition of insulin-stimulated tyrosine phosphorylation of insulin receptor and of its substrates (Fig. 2); these include the proteasome-mediated degradation, phosphatase-mediated dephosphorylation, and kinase-mediated serine/threonine phosphorylation [71].

It has been clearly documented by human and animal studies that both acute exercise [72] and physical training are [73] capable to ameliorate the molecular abnormalities that are responsible of the insulin resistance, contributing in this way to restore the physiological insulin sensitivity. Notably, acute exercise and physical training ameliorate the insulin signaling through different molecular mechanisms.

## Acute Exercise and Insulin Signaling

**Fig. 3:**
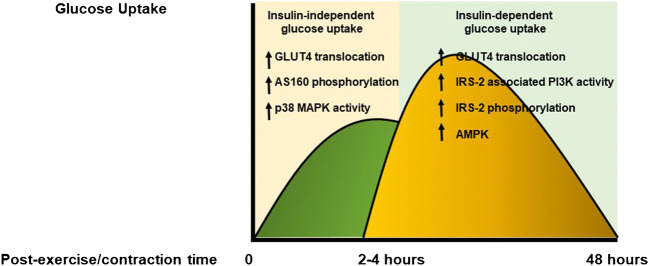
Effects of acute exercise on glucose uptake and insulin signaling. Acute exercise evokes an increase of glucose uptake which is time-dependently regulated by insulin-independent and insulin-dependent mechanisms. The exercise-induced enhanced insulin sensitivity is mediated by different pathways that work in parallel with the canonical insulin signaling cascade. AMPK: AMP-activated protein kinase; AS 160: AS 160 protein; GLUT4: insulin-responsive glucose transporter; IRS: insulin receptor substrates; PI3K: phosphoinositide 3-kinase

The mechanisms that increase the acute exercise- and/or muscle contraction-induced glucose uptake are regulated by insulin-independent and insulin-dependent pathways in a time-dependent fashion. In addition, the enhanced insulin sensitivity following exercise is not mediated by the activation of the classic insulin signaling cascade (Fig. 3).

### Studies in Animals

The molecular mechanisms that account for the increased insulin signaling following exercise were defined by the pioneering studies in rodents, particularly, in genetically engineered mice. These studies showed that the increase of muscle glucose uptake detected immediately after exercise is mediated by a PI3K-independent recruitment of GLUT4 on the plasma membrane [74, 75]. In addition, it was found a close relationship between the exercise-induced translocation of GLUT4 on the cell surface and glucose uptake [76]. In the skeletal muscle of transgenic mice lacking the gene encoding for the IR, it was reported that exercise increases the 2-deoxy-glucose uptake, and that this phenomenon is not amplified by insulin [77]. It has also been reported that acute exercise enhances the activities of the members of the mitogen-activated protein kinases (MAPKs) [78]. The MAPK family include three different groups of serine/threonine kinases that can be separated into three major subtypes: the extracellular signal-regulated kinases 1/2 (ERK1/2), the p38 MAPK, and the c-jun NH2 terminal kinase (JNK). It has been reported that the stretch-stimulated glucose uptake in isolated and perfused skeletal muscle was reduced by the inhibition of p38 MAPK, while it was not affected by PI3K inhibition [79]. In addition, it has been also demonstrated that the substrate of Akt: the AS 160 protein, which is involved in the translocation of the GLUT4 on cell membrane, plays a key role in the regulation of glucose uptake immediately after exercise, through an insulin-independent mechanism [80]. Altogether these data indicate that the increase of muscle glucose uptake recorded immediately after exercise and/or contraction is an insulin-independent phenomenon, and the mobilization of GLUT4 from different intracellular pools represents the rate-limiting mechanism.

Subsequently, for up to 48–72 h, insulin signaling is enhanced. In particular, it has been reported that in this phase, insulin-induced activation of PI3K is markedly increased [81]. However, this effect is mediated neither by enhanced IRS-1 tyrosine phosphorylation, nor by the increase of the interaction between IRS-1 and PI3K. This indicates that alternative molecular pathways account for the exercise-induced increase of insulin signaling. In this regard, Howlett KF et al. [82] evaluated whether the post-exercise insulin-induced increase of PI3K activity is an IRS-2-dependent phenomenon. In basal conditions, insulin stimulation, in control mice (wild type, WT), increases both tyrosine phosphorylation of IRS-2 and IRS-2-associated PI3K activity, and that exercise amplifies by 3.5-fold and by 5-fold the insulin-stimulated tyrosine phosphorylation of IRS-2 and IRS-2-evoked PI3K activity, respectively. On the contrary, in IRS-2 knockout mice, the additive effects of exercise on insulin modulation of IRS-2 and PI3K activity were blunted. These results support the concept that IRS-2 can partially account for the insulin-evoked increase of PI3K activity after exercise. However, it must be considered that the increase of insulin sensitivity following acute exercise is a complex and multifactorial process, which involves different molecules. Several data indicate that AMP-activated protein kinase (AMPK) plays a role as co-factor for the increase of insulin sensitivity following acute exercise. In this regard, it has been reported that skeletal muscle contraction enhances both insulin-evoked glucose uptake and AMPK phosphorylation/activation [83, 84]. The mechanistic role of AMPK in the regulation of insulin sensitivity has been corroborated by the study of Kjøbsted et al. [85] who demonstrated that stimulation of WT mice with an activator of AMPK, the 5-amino-4-imidazoleacarboxamide riboside (AICAR), increases insulin-mediated glucose uptake in the skeletal muscle, and this effect is blunted in transgenic mice with reduced or ablated AMPK activity in the skeletal muscle, suggesting that AMPK is necessary and also sufficient to improve insulin sensitivity.

In addition, it has been demonstrated that acute exercise ameliorates insulin signaling also in the presence of insulin resistance. In fact, one single session of exercise is able to improve both insulin-induced glucose uptake and insulin signaling in rodents fed with high-fat diet [86, 87], which is an established model of insulin resistance [88].

### Studies in Humans

Several evidences show that a single bout of exercise is capable to increase insulin-mediated glucose uptake in healthy subjects [89], as well as in subjects with insulin resistance [90, 91]. The analysis of signaling obtained by muscle biopsies showed that the increased insulin effect after acute exercise is not caused by the enhancement of the early steps in the insulin cascade. However, it has been reported that distal downstream kinases of the insulin signaling pathway are activated earlier than the proximal signaling molecules [92]. At this regard, it has been documented [93] in healthy subjects that acute exercise improves lipid-induced insulin resistance by increasing TBC1D4 signaling, which, in turn, promotes the translocation of GLUT4 on the cell membrane. Interestingly, TBC1D4 belongs to a family of multikinase substrates that contains the phosphorylation domains for Akt and AMPK. This allows to speculate that alternative pathways account for the beneficial effects of exercise on insulin sensitivity, likely acting downstream its receptor.

Altogether these studies demonstrate that acute exercise improves the insulin signaling and skeletal muscle glucose uptake. Although they allow to hypothesize that acute exercise reverts the molecular basis involved in the pathogenesis of insulin resistance, it is noteworthy that the restored insulin sensitivity is not mediated by the improvement of the early steps of the classic insulin signal cascade. Thus, it is reasonable to assert that acute exercise improves insulin signaling through different pathways that work in parallel or distal to the molecular defect.

## Exercise Training and Insulin Signaling

The molecular mechanisms accounting for the improvement of insulin sensitivity induced by exercise training are complex and still not completely defined.

### Studies in Animals

In rats, exercise training increases the gene expression of IR, IRS-1, and PI3K [94], as well as the GLUT4 expression [95, 96] and its translocation to the plasma membrane. In addition, in response to different intensities of exercise training, a close relationship is observed between GLUT-4 expression and insulin-stimulated muscle uptake [97, 98]. The mechanisms accounting for the exercise training-induced upregulation of GLUT-4 expression and its translocation on the plasma membrane include several intracellular messengers that are activated by different exercise-induced mechanical and metabolic stimuli. AMPK plays a key role to enhance insulin-induced glucose uptake in response to acute exercise by recycling the existing GLUT4 molecules to the plasma membrane; additionally, sustained activation of AMPK can increase the expression of GLUT4 transporters in the skeletal muscle [99] by enhancing its transcription. This phenomenon may be mediated by the upregulation of the expression of peroxisome proliferator-activated receptor gamma coactivator 1alpha (PGC-1alpha) [100], which has been demonstrated to regulate the expression of GLUT-4. In addition, the stretching of muscular fibers induced by the contractile activity plays a role in the regulation of MAPK activity. Exercise training activates all types of MAPKs in the skeletal muscle of rats [101, 102]; however, the upstream mediators and the downstream targets of MAPKs are not yet clearly identified.

Since pro-inflammatory cytokines as well as oxidative stress play a key role in the impairment of insulin signaling and in the increase of CV risk [103], an additional mechanism that account for the improvement of insulin signaling in response to exercise training is the reduction of inflammation as well as the oxidative stress. Studies performed in animal models of insulin resistance have documented that exercise training is capable to blunt the enhancement of pro-inflammatory cytokines such as IL-6 and TNF-α and, at the same time, ameliorates the anti-inflammatory response by increasing the circulatory levels of IL-4 and IL-10 [104, 105], resulting in a decrease of CRP levels [106, 107]. Therefore, the exercise training-induced reduction of inflammatory status contributes to improve the insulin signaling.

### Studies in Humans

In humans, the exercise training enhances the IRS-1-mediated activation of PI3-K [108]; however, this phenomenon is not mediated by the increase in gene expression of components of the early steps of insulin cascade [109]. This suggests that exercise training improves the efficiency of insulin pathway without affecting the protein expression, or alternatively ameliorates insulin sensitivity by increasing post-receptor signaling. In this regard, it is noteworthy that in diabetic subjects, exercise training does not enhance the ability of insulin stimulation to increase IRS-1-mediated PI3K activity, but increases GLUT4 protein expression by 22%, and this phenomenon is associated by enhanced Akt protein expression [110]. To note that Akt phosphorylation inhibits the GSK-3β activity, this promotes in the stimulation of gene transcription and protein synthesis. Thus, it is reasonable to hypothesize that the improvement of insulin sensitivity in response to chronic to exercise is mainly induced by the regulation of transcriptional processes.

Exercise training is associated with an anti-inflammatory response [111]. However, there are still conflicting results about the type and intensity of exercise needed to achieve an anti-inflammatory effect. In fact, it has been demonstrated that low to moderate intensity of exercise training has no effect on circulating levels of pro-inflammatory mediators [112, 113], while high-intensity exercise aerobic training decreases circulating levels of IL-6 [114]. Further studies demonstrated that the combination of aerobic and resistance exercise training confers a more efficacious anti-inflammatory action characterized by a decrease of circulating levels of pro-inflammatory molecules, associated with an increase of anti-inflammatory molecules such as IL-4 and IL-10.

There are still undefined results about the capability of the exercise training to modulate the levels of the adiponectin, which is an insulin-sensitizing cytokine produced by adipose tissue. Simpson and Singh [115] in a meta-analysis that evaluated 33 studies found only a weak effect of exercise prescription on the adiponectin plasma levels; in fact, the increase of serum adiponectin was detected in 38% of randomized controlled trials. However, it was found that the combination of exercise training and hypocaloric diet increased the expression of adiponectin receptor in the skeletal muscle [116]. Altogether these studies indicate that exercise training influences the oxidative homeostasis by increasing the resistance to the oxidative stress, and at the same time, by reducing the exposure to the oxidative stress.

The improvement of insulin sensitivity induced by the exercise training, rather than the amplification of canonical signal transduction cascade, seems to be mediated by the activation of transcription factors, which in turn regulate the expression/suppression of target genes, which ultimately modify the metabolic asset of the cells. Further investigations are required to better clarify the molecular basis of the exercise training-evoked improvement of insulin signal.

Acute exercise and physical training differently modulate the insulin sensitivity; the mechanisms that account for this phenomenon are resumed in Table 1.

**Table 1:** The mechanisms that account for acute exercise and physical training which differently modulate the insulin sensitivity

	Acute exercise	Exercise training
Whole body glucose tolerance		
Post-exercise OGTT	↔	↑
Post-exercise meal	↑	ND
Whole body glucose disposal	↔	↑
Insulin-stimulated skeletal muscle glucose transport	↑	↑
GLUT-4 translocation	↑	↑
GLUT-4 expression	↔	↑
Insulin-stimulated IR		
Protein expression	ND	ND
Tyrosine phosphorylation	↑	ND
Insulin-stimulated IRS-1/2		
Protein expression	ND	ND
Tyrosine phosphorylation	↑	ND
Insulin-stimulated PI3K		
Protein expression	ND	ND
Activity	↑	↔
AKT		
Protein expression	ND	↑
Serine phosphorylation	ND	↔

*IR* insulin receptor, *IRS* insulin receptor substrates, *ND* not determined, *OGTT* oral glucose tolerance test, *PI3K* phosphoinositide 3-kinase

## Clinical Implications and Future Directions

The beneficial effects of exercise training in CV prevention are evidence-based documented and have robust pathophysiological and molecular basis. The scientific societies, for the primary and secondary CV prevention, highly recommend to practice at least 150 min/week of moderate-intensity aerobic physical activity, or 75 min/week of high-intensity activity [117, 118]. Unfortunately, in the real life, only 30–40% of subjects at high risk are adherent to this advice [119–121]. Therefore, the promotion of the exercise training represents the current and future challenge of the healthcare systems for the improvement of CV prevention. At this regard, the use of innovative and/or integrated technologies like *telemedicine* can help to overcome the cultural, environmental, and psychological barriers that limit the adherence to the exercise programs. In addition, the new technologies (wearable monitors, mobile apps, etc.) will be able also to detect the changes of metabolic and hemodynamic parameters evoked by exercise training [122, 123]. These data can be shared with general practitioners and specialists contributing to improve the CV prevention. The development of these integrated strategies for CV prevention, nowadays, is fully supported by public healthcare systems.

A point that also needs to be elucidated is how optimize the beneficial effects of exercise on insulin sensitivity (i.e., timing of the day, disease specificity, intensity, frequency). At this regard, it has been reported by Savikj et al., in a cross-over study [124], in patients with type 2 diabetes, that afternoon high-intensity interval training (HIIT) was associated with a better glucose control compared with morning HIIT. This study underlines that the timing of exercise plays a role, at least, in the improvement of the insulin sensitivity in diabetes. Since obesity, hypertension, MS, and dyslipidemias can be considered different phenotypes of insulin resistance, further studies are needed to clarify if the benefits of the timing of the exercise can be extended also to these disturbs.

Finally, it must be highlighted that, although it has been proven a curvilinear relationship between the amount of physical activity and CV protection, it is also true that high doses of strenuous exercise increase the CV risk [125, 126]. Thus, it has been hypothesized that this relationship rather than to be curvilinear is U- or J-shaped. This is still a controversial issue that needs to be further clarified. In particular, so far, in normal subjects has not been identified any upper limit for moderate-intensity exercise; however, it has been demonstrated that higher amount than 100 min a day does not confer any additional CV protection [127]. On the other hand, high doses of strenuous exercise among the subjects with an overt CV disease have been found to be associated with the increased incidence of CV adverse events. To resolve this issue, it is reasonable to use the changes of insulin sensitivity as a maker to personalize the amount of physical exercise that confers CV protection. Studies in this direction will further support the role of enhanced insulin sensitivity as mediator of beneficial effects of exercise in CV prevention.

## Abbreviations

AICAR: 5-Amino-4-imidazoleacarboxamide riboside
AMPK: AMP-activated protein kinase:
BP: Blood pressure
CRP: C-reactive protein
CV: Cardiovascular
cGMP: Cyclic guanosine mono-phosphate
DBP: Diastolic blood pressure
eNOS: Endothelial nitric oxide synthase
GSK-3: Glycogen synthase kinase 3
HbA1c: Glycosylated hemoglobin
HDL: High-density lipoprotein
HOMA: Homeostatic model assessment–insulin resistance
IGT: Impaired glucose tolerance
IL: Interleukine
IR: Insulin receptor
IRS: Insulin receptor substrates
GLUT4: Insulin-responsive glucose transporter
ICAM: Intercellular adhesion molecule
LTPA: Leisure-time physical activity
LDL: Low-density lipoprotein
MS: Metabolic syndrome
MAPKs: Mitogen-activated protein kinases
NO: Nitric oxide
NAFLD: Non-alcoholic fatty liver disease
PGC-1alpha: Peroxisome proliferator-activated receptor gamma coactivator 1alpha
PI3K: Phosphoinositide 3-kinase
SBP: Systolic blood pressure
TNF: Tumor necrosis factor
